# Threat Modeling—How to Visualize Attacks on IOTA?

**DOI:** 10.3390/s21051834

**Published:** 2021-03-06

**Authors:** Ikram Ullah, Gerard de Roode, Nirvana Meratnia, Paul Havinga

**Affiliations:** 1 Pervasive Systems Group, Department of Computer Science, University of Twente Enschede, 7522 NB Enschede, The Netherlands; n.meratnia@utwente.nl (N.M.); p.j.m.havinga@utwente.nl (P.H.); 2 Faculty of Electrical Engineering, Mathematics and Computer Science, University of Twente, 7522 NB Enschede, The Netherlands; g.deroode@student.utwente.nl

**Keywords:** IoT, blockchain, IOTA, decentralization, vulnerabilities, CVSS v3.0

## Abstract

Internet of Things (IoT) has been deployed in a vast number of smart applications with the aim to bring ease and comfort into our lives. However, with the expansion of IoT applications, the number of security and privacy breaches has also increased, which brings into question the resilience of existing security and trust mechanisms. Furthermore, the contemporaneous centralized technology is posing significant challenges viz scalability, transparency and efficiency to wide range of IoT applications such as smart logistics, where millions of IoT devices need to be connected simultaneously. Alternatively, IOTA is a distributed ledger technology that offers resilient security and trust mechanisms and a decentralized architecture to overcome IoT impediments. IOTA has already been implemented in many applications and has clearly demonstrated its significance in real-world applications. Like any other technology, IOTA unfortunately also encounters security vulnerabilities. The purpose of this study is to explore and highlight security vulnerabilities of IOTA and simultaneously demonstrate the value of threat modeling in evaluating security vulnerabilities of distributed ledger technology. IOTA vulnerabilities are scrutinized in terms of feasibility and impact and we have also presented prevention techniques where applicable. To identify IOTA vulnerabilities, we have examined existing literature and online blogs. Literature available on this topic is very limited so far. As far as we know IOTA has barely been addressed in the traditional journals, conferences and books. In total we have identified six vulnerabilities. We used Common Vulnerability Scoring System (CVSS v3.0) to further categorize these vulnerabilities on the basis of their feasibility and impact.

## 1. Introduction

In the current era of Internet of Things (IoT) revolution, the overwhelming applications of IoT are evolving in many domains [1,2]. The amount of data IoT devices generate is rapidly increasing [3]. Moreover, the relevance and importance of big data is growing fast, data is constantly being collected and analyzed, leading to innovation and economic growth [4]. The increase in data generation is directly proportional to the increase in number of IoT devices. Such an increase can raise questions regarding transparency, liability, privacy, trust and security. IoT devices are required to transfer data and value in a sufficiently private and secure manner. Traditional centralized technologies are challenged to meet the rapidly increasing demands of IoT and to respond to concerns such as mentioned above. Moreover, centralized technologies themselves face security and trust challenges like “central point of failure” and “third party involvement”. Currently, value transfer depends largely on centralized technologies. For example, existing banks’ ledgers used by private or government banks, which include financial records are trusted without any transparency nor the ability to verify the data or insight in how it is secured. Such delegated and unverifiable trust is posing a major obstacle for an inclusive and permissionless economy. Next to the trust aspect, new technologies for data and value transfer should aim to achieve security, a sufficient level of privacy and low cost to be suitable for IoT applications. An initial shift towards trustless systems of data and value transfer is made in the rise of blockchain-driven decentralized cryptocurrencies, such as Bitcoin [5,6,7]. In these systems, third party intermediaries become obsolete because they are replaced by a protocol and cryptography, which results in decentralized and distributed trust. Technologies which aim to eliminate the centralized trust factor in transfer of data or value, such as the above example, often use Distributed Ledger Technology (DLT) to store data.

Technology architectures are rapidly undergoing fundamental changes to become compatible with IoT requirements [8]. Contrary to centralized technology, decentralized technology allows to form distributed, secure and permissionless storage and transfer of data and value. The technological advantages of DLTs are: durability, transparency, immutability, process integrity and security. DLTs use cryptographic algorithms to override the need for trusted third parties in online commerce or any application domains. DLTs are practically implemented in many applications and solutions. For example, privacy aware decentralized solutions are proposed [4,9], decentralized identity management [10], electronic medical records [11], smart vehicle charging stations [12] and intelligent transportation systems [13].

As the development of DLTs is still underway, many different distributed technologies, such as blockchain, Hashgraph, IOTA and others, are proposed. IOTA is a permissionless distributed ledger that utilizes a novel invention, called the “tangle”, at its core [14]. It is also implemented in a homonymous cryptocurrency with no transaction fees. IOTA is presented as a faster, cheaper and more secure technology to achieve trust [15]. Existing IOTA applications like [12] demonstrate that it indeed has real-world value and potential. IOTA works quite different compared to blockchain and other DLTs and achieves a range of unique features: scalability, decentralization, no transaction fees and quantum immunity [16]. Therefore, IOTA has attained huge interest by many. However, increased adoption of IOTA also raises the need for proper security evaluation of the employed technology. The purpose of this study is to present a new way to explore and visualize the security challenges of DLTs, which is specifically applied to IOTA. Similar to any other technology, DLTs are not immune to every security threat. Through this study, a contribution is made to the methods that can be employed to understand and explore security challenges of DLTs like IOTA. This contribution consists of a novel way of looking at security threats by applying threat modeling. State of the art research work is conducted to investigate the DLT security challenges [17,18]. According to the best of our knowledge and investigation, no dedicated research is conducted that employs threat modeling to evaluate IOTA vulnerabilities. In this research, Common Vulnerability Scoring System (CVSS) v3.0 [19] threat modeling is employed to evaluate IOTA security vulnerabilities.

### 1.1. Our Contribution

In this work, the existing literature is explored to identify known possible IOTA vulnerabilities and security issues and to give a coherent and formal overview of the found IOTA security issues. Furthermore, in order to classify the identified vulnerabilities based on their severity and impact, CVSS v3.0 based threat modeling is used. The six IOTA vulnerabilities we have identified are: Curl-P hashing, replay attack, double spending, splitting attack, 34% attack and necessity of an “assiduous honest majority” and centralization.

### 1.2. Organization

This paper is organized as follows. Section 2 introduces and compares blockchain and IOTA. Section 2.2.3 describes the unique security aspects of IOTA. Section 3 thoroughly discusses the identified IOTA vulnerabilities. Section 4 considers the feasibility of exploiting the identified vulnerabilities. Section 5 discusses the threat modeling methods and findings. Section 6 analyses the executed threat model.

## 2. Distributed Ledger Technologies

In this section two different DLTs, Blockchchain and IOTA are studied and compared.

### 2.1. Blockchain

**Figure 1:**
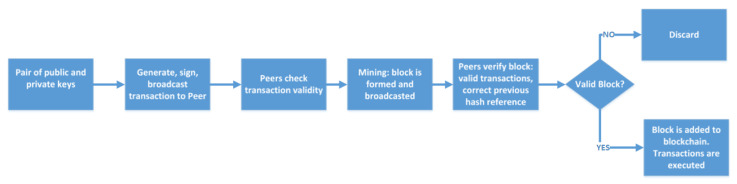
How the blockchain works.

**Figure 2:**
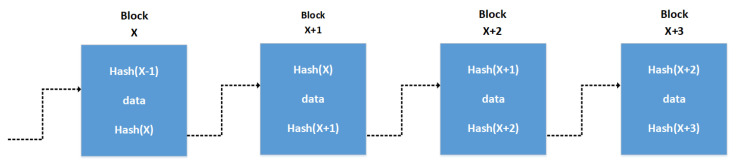
Blockchain visualization, arrows represent links between the blocks.

“Blockchain is essentially a distributed database of records or public ledger of all transactions or digital events that have been executed and shared among participating parties” [20]. A possible generic blockchain working mechanism is shown in Figure 1. Blockchain technology was first introduced with Bitcoin to solve double spending problem [7]. Blockchain enables trustless networks, where the parties can transact even though they do not trust each other. In blockchain, a block is a set of transactions or records and the chain is linking blocks together as shown in Figure 2. Each block in the blockchain consists of block number, hash of previous block, nonce, data (transactions), timestamp and the hash of the current block [21]. Nonce is a random number used in proof of work (PoW), which is discussed later in the paper. The hash is important for ordering of transactions, it links the blocks in an ordered chain since the hash of the previous block is included in the next block [22]. Any node that solves a difficult puzzle in order to form a new block is called a miner. The miner uses previous block information and new transactions to form a block and add it to the blockchain. A blockchain can be public or private; in public blockchain, nodes do not require permission to write or read data on the blockchain. Conversely, in private blockchain the nodes are known and authenticated before reading and writing.

Smart contracts, which are small computer programs that execute unambiguously on all network nodes, can be used to automatically perform transactions when certain conditions set in the code are met. The significance of blockchain and smart contracts is that manual contracts can now be implemented digitally in code which is executed by the blockchain network. Since smart contracts are stored on the blockchain, they are transparent and cannot be altered or deleted [23].

Transactions in a public ledger are verified through consensus among the participants [24]. Consensus on the transactions and the order of transactions is important in order to maintain an unambiguous distributed ledger among all the nodes. Distributed consensus among the participating nodes is achieved through consensus algorithms. Some of the consensus algorithms used in DLTs are proof of work (PoW) [25], proof of stake (PoS) [25] and Practical Byzantine fault tolerance (PBFT) [25].

#### 2.1.1. Blockchain Applications

Bitcoin [7] is the first realization of Blockchain technology. Blockchain has the potential across many application domains. Some of the prominent blockchain applications are: cryptocurrency [5], voting system [26], smart contracts [27], eliminate counterfeit drugs [28], identity management [29], privacy preserving solutions [30] and health care [31].

#### 2.1.2. Blockchain and IoT

Mostly, IoT devices have limited power and computational resources and hence are more susceptible to malicious attacks. IoT devices are vulnerable to wide span of security attacks [32,33] and trust attacks [34]. Security related attacks against IoT devices as mentioned in [32] are: eavesdropping, traffic analysis, camouflage, node capture, sybil and denial of service. Similarly, in IoT ecosystems any malicious devices can perform trust related attacks such as self promotion [34], bad-mouthing [34], ballot stuffing [34], opportunistic service [34] and on-off trust related attacks [34]. IoT and blockchain integration can potentially exploit all the unique characteristics of blockchain technology, unerringly vanquish IoT challenges and develop verifiable IoT networks [35]. Integrating blockchain and IoT can potentially provide a new way to create value and transform various industries [36].

#### 2.1.3. Blockchain Limitations

Blockchain has instigated substantial advantages but not without ineluctable downsides [37,38,39,40,41,42,43]. The notable downside of blockchain is the transactions fees paid for transactions to miners. Transactions fees are posing challenges to the rapidly evolving IoT industry where the importance of micropayments is increasing. However, paying transaction fees larger than the transaction amount is not economical [14]. Moreover, blockchain poses challenges to IoT because of the limited throughput, high latency and power consumption nature of blockchain.

### 2.2. IOTA

IOTA is a DLT that uses the tangle as a data structure, which is based on directed acyclic graph (DAG), to store its ledger. Due to this unique architecture, IOTA has no blocks, no chain and no miners. Therefore, IOTA work quite differently compared to blockchain and other DLTs. For example, consensus in IOTA is procured differently and transactions are executed in another way. However, there are still some underlying similarities between blockchain and IOTA; both of these technologies employ distributed databases, peer-to-peer networks [44,45], and both are utilizing consensus mechanisms. Still, IOTA’s DAG architecture disburses a range of distinctive features: scalability, decentralization, fast transactions, no transaction fees, quantum immunity, data integrity and interoperability with blockchain [16,46].

**Figure 3:**
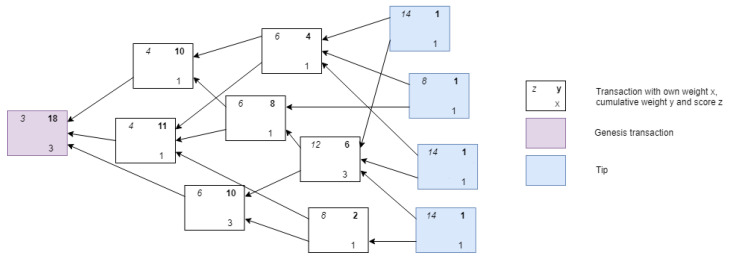
Weight, cumulative weight and score in the tangle.

**Figure 4:**
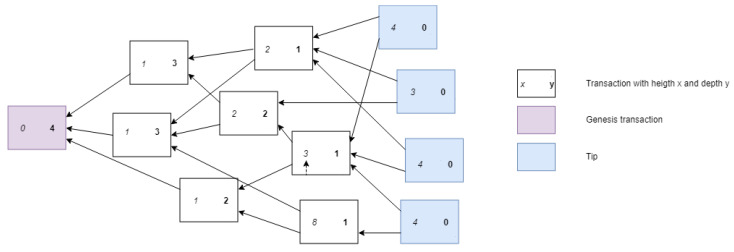
Height and depth in the tangle.

The IOTA network consists of nodes, which issue and validate transactions. Validated transactions are then added to the tangle. In the tangle, which is a Directed Acyclic Graph (DAG), vertices represent transactions and edges represent approval of transactions. Each of the vertices in the graph has some metrics associated with it; weight, cumulative weight, height, depth and score. The weight corresponds to the amount of work done by the transaction issuing node. The idea of weight is used to avoid spamming and other attack vectors; it is assumed that no entity can generate large number of transactions with acceptable weight in short time period [14]. The cumulative weight of a transaction is the sum of the own weight and all the own weights of the transactions that (in)directly approve the transaction. As said, approval in the tangle is represented by the edges. For instance, transaction A directly approves transaction B if there is a directed edge between them, while A indirectly approves B if there is a directed path of length at least two between them. Each site in the graph also has a height which is the maximal distance to the genesis site via some directed path, and each site has a depth, the maximal distance to a tip via some reverse directed path. The genesis transaction is the transaction that started the directed acyclic graph and in which all the tokens are initialized. It does not approve any transactions but is referenced by all transactions. Finally, a site also has a score, which is the sum of its own weight and the own weights of all the sites that it (in)directly approves. The more a transaction is approved, the higher the weight, the higher level of confidence it achieves [14]. Figure 3 and Figure 4 visualize these metrics in an example tangle.

For a node to issue a transaction, it performs computational work to approve other transactions, in such way every node of the network contributes to the security and validity of the tangle. The work is done to ensure that the transactions are not conflicting. When conflicting transactions are encountered, nodes should use the tip-selection algorithm to decide which of the transactions should be accepted. Given multiple runs of the tip-selection algorithm, the transaction that is more likely to be selected by the algorithm should be accepted [14].

#### 2.2.1. IOTA Background Knowledge

Some of the terminologies used in IOTA are briefly described below:

##### Transaction

**Figure 5:**
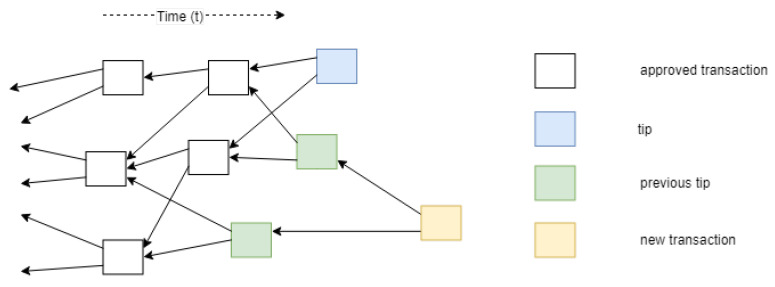
Adding a transaction to the tangle.

A transaction in IOTA consists of many values such as; unique hash of the transaction, output address (recipient), input address (source), transfer value, timestamp, bundle hash, trunktransaction (first transaction approved), branchtransaction (second transaction approved) [47]. Trunktransaction and branchtransaction are two randomly selected transactions which are required to be validated before a transaction can be added to the tangle. This process of validating previous transactions and adding the current transaction is equivalent to executing a transaction. A new transaction that has not been approved by any other transaction yet is called a tip as shown in Figure 5.

##### Trinary

Binary logic is commonly used in computing systems for data representation. In binary logic data is represented as bits and bytes. A bit can hold either 1 or 0 value and a byte consists of 8 bits. On the contrary, Trinary (ternary) logic is based on trits and trytes. In balanced trinary, a trit can possibly hold 1, 0, or −1 value and a tryte is made up of 3 trits. Thus trytes can hold 33 possible values. IOTA uses balanced trinary logic, which is then converted to binary for the purpose of compatibility with binary devices. Trinary is used because trinary arithmetic is found to be more efficient and logical than binary logic [46,48] and an important numeral system in the future of computing.

##### Private Keys

A seed is a random set of trytes, used to access an account and funds. Addresses are generated from private keys, which in turn are derived from the seed. Private keys are generated on the client side in the browser or on an offline computer [47]. IOTA uses Winternitz one-time signatures. Private key and address should not be reused. Consecutive reuse of private keys can lead to forging signature and hence loss of funds [47].

##### Bundle

IOTA uses the concept of transaction bundles—a bundle is a set of input and output transactions. Inputs are the source addresses and amounts to be transferred from them, outputs are the destination addresses and the amounts to be transferred to them. Although the number of transactions in a bundle is not limited, they consist normally mostly of four transactions [49], an output, an input, a signature and a remainder output. All the transactions in a bundle must get accepted by the network together; in that sense, a bundle is atomic [47,49].

##### Consensus

Consensus entails that all the nodes are agreed on one state of the ledger. From security and performance perspective, tip selection strategy is the most important aspect in tangle-based cryptocurrency [14]. This is because consensus will ultimately be based on tip-selection, and therefore most of the attacks are aimed at exploiting the tip-selection strategy. In order for a transaction to be confirmed and validated, IOTA establishes consensus using one of the following two methods as per the IOTA whitepaper version 1.4.3 [14,50]: the Coordinator (centralized approach) and Markov chain Monte Carlo (MCMC) tip selection algorithm (distributed and probabilistic approach). The Coordinator is an IOTA network node operated by the IOTA foundation. It issues zero-valued milestone transactions at some rate [50]. Transactions that are referenced by milestone are considered as confirmed, while transactions not referenced by milestone are not confirmed. The MCMC algorithm is a probabilistic approach to reaching consensus, which is planned to eventually replace the Coordinator. The algorithm uses random walkers that will walk towards tips from a given depth in the tangle. Probability metrics dependent on cumulative weight determine the pathing choices of the walker to some random tip. Consensus over conflicting transactions can now be achieved by running the MCMC tip selection algorithm multiple times to discover which of the conflicting transactions is more likely to be (indirectly) approved by the selected tips.

##### Proof of Work

Proof of work (PoW) is a resource-intensive task that must be performed before issuing a transaction. Validating the integrity of PoW is easy and fast. Minimum Weight Magnitude (MWM) represents the number of required trailing zeros in transaction hash. The difficulty of PoW depends on MWM. In PoW, nonce is calculated to achieve trailing zeros as specified in MWM. A nonce is valid, when a hash generated by PoW contains the correct number of zeros as required in MWM. A valid nonce is required for the transaction to be accepted by the tangle network. PoW is performed for three purposes: Distributed denial-of-service prevention, immutability and to prevent double spending [51]. PoW in IOTA is less computationally expensive than PoW employed in miner-based ledgers such as Bitcoin [52]. For PoW, IoTA uses CURL algorithm which is developed by IOTA foundation.

#### 2.2.2. IOTA Applications

IOTA cryptocurrency is designed specifically for the IoT industry [14]. This is because it is a faster, cheaper and more secure technology to achieve trust [15], hence suitable for IoT ecosystem and to trounce IoT security and trust challenges mentioned earlier. By utilizing IOTA’s potential features, many use cases which were not possible before can now be realized—for example, real-time streaming payment services for data and energy, immutable data history tracking for supply chains and computational resource sharing [46]. As IOTA is designed for tiny devices with limited resources and offline computation, it enables new business models which could not be achieved with traditional technologies or blockchain.

#### 2.2.3. IOTA Security

Security is crucial for cryptocurrencies. Nevertheless, IOTA endured very complicated security challenges and vulnerabilities; it has frequently been regarded as insecure [53]. There have been a few short reports describing possible vulnerabilities in IOTA, and the IOTA whitepaper itself also addresses possible attacks on IOTA [14,54,55,56,57].

## 3. IOTA Vulnerabilities

The aim of this section is to give a clear overview of the potential security issues of the IOTA cryptocurrency and the immediate threats they constitute. Six claimed IOTA vulnerabilities, and potential attacks if exploited, which were found in literature and various online sources are elaborated. If useful, visualizations and mathematical formalizations will be provided. Where available, IOTA foundation response to these vulnerabilities thus far is also elaborated.

### 3.1. Curl-P Hashing

IOTA implemented Curl-P hashing algorithm for things like transaction hashing and signatures. A team from Massachusetts Institute of Technology (MIT) found potential vulnerabilities in the security of IOTA by analyzing this hashing function. In September 2017 they published a report and accompanying article [53,54] outlining possible attacks on IOTA through using hashing collisions in Curl-P algorithm. Using differential cryptanalysis, they were able to find collisions in the algorithm [58].

Both of the attacks are based on the following series of events:

1. attacker creates two bundles that collide; which hash to the same value.
2. attacker asks victim to sign of the bundle
3. victim signs the bundle and attacker gets the signature of that bundle
4. attacker couples the signature to the other bundle he generated, and attacker publishes the bundle with the victim’s signature to the network

#### 3.1.1. Waste Money Attack

In a waste money attack the attacker sends the bundle to the victim. The bundle contains a transaction that sends IOTA’s from the victim to a given address. The victim then signs this bundle, and the attackers take the signature off the bundle and couples it to another bundle which contains the same transaction, only the address to which the victim now pays out is slightly different. The attacker sends this bundle out into the network and then tells the victim that they paid to the wrong address and need to pay again. The funds the victim lost to a wrong address are now wasted [54].

#### 3.1.2. Steal Money Attack

In a steal money attack the attacker sends the bundle to the victim. The bundle contains a transaction that sends a certain amount of IOTA from the victim to some address. The victim then signs this bundle, and the attacker takes the signature of bundle and couples it to another bundle which contains a different transaction, to the same address but with a different amount. This second bundle is then sent out into the network, spending more or less IOTA from the victim instead of the amount originally signed by the victim. Thus, the attacker could increase the amount the victim pays them and in that way steal IOTAs [54].

**Figure 6:**
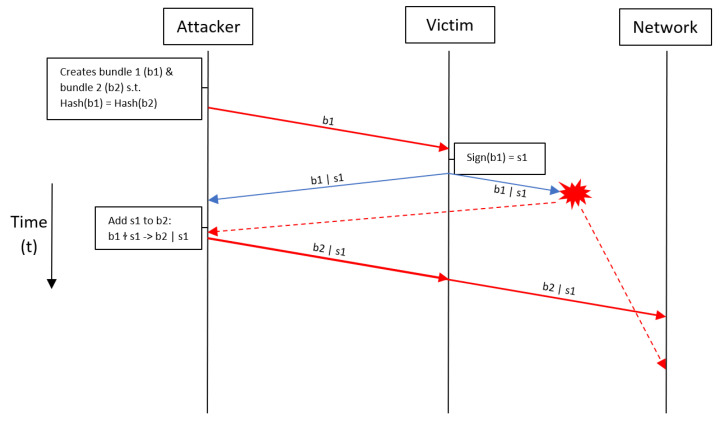
Waste and steal money attack.

Figure 6 visualizes the procedure of the waste and steal money attacks. The red “star” symbol stands for the necessity that the attacker outruns the victim. Thus, either the attacker did an eclipse attack, in which case the bundle that was sent out into the network ends up at the attacker, which is the dotted line to the attacker, or the victim was somehow much slower than the attacker. In that case the victims bundle and signature packet arrives much later in the network than the attacker’s fraudulent packet which is represented by the dotted line to the network. For the attack to be successful, the malicious bundle has to “outrun” the original transaction bundle. That means that either the attacker needs a lower confirmation time for its transactions, and thus lower propagation time, or the attacker needs to do an eclipse attack [59]. An eclipse attack is an attack in which the attacker limits the connectivity of the victim to the network. All the traffic that the victim sends out is routed via the attacker or blocked by the attacker. The attacker controls the connections the victim has with the network and thus decides what messages the victim receives/can send [5]. Because it is not very likely that all these things will happen, the victim signing a foreign bundle and the attacker being able to “outrun” the victim, it is claimed this is an unlikely attack [59].

After the possibility of generating collisions was made known to the IOTA team, they initially responded with updating their hash function to Keccak/SHA-3 [60]. Later this became Kerl, which is Keccak-384, and the IOTA foundation responded to attack publication [54] with a blog [59]. They also did not confirm that the hashing collisions would cause a vulnerability to attacks but argued that the waste money and steal money attacks are extremely unlikely to occur. They explain that it would require the victim to be naïve and sign bundles that are not his own. The showed attacks are based on a “chosen message attack” and not on “known message attack”. This means that the attacker first creates a message, or in this case a bundle, that the victim then has to sign. When the attacker gets back the signed bundle they must extract the signature and use it on a different bundle, which they then have to send out into the network. In case of a known message attack, the attacker would only have to know the message, or in this case bundle, of the victim and can then do the attack. This is more powerful, because that attack has a wider application. Furthermore, the IOTA foundation responded to vulnerability report [54] and accompanying blogpost [53] with a series of blogposts [61,62,63,64]. In these blogposts, a response is given to each of the issues that are addressed in the vulnerability report. About the collisions in Curl-P it is said that it was known that Curl-P had collisions and that this was intentional. They claim that the colliding hash function functioned as a copy protection mechanism and that IOTA was not vulnerable to attacks exploiting these collisions because the Coordinator offered protection against such attacks [64]. The Curl-P hash function is still used in IOTA for some specific purposes [65]. It is unknown whether this causes any further vulnerabilities.

### 3.2. Replay Attacks

A different way to steal funds from addresses is by “replaying” transaction bundles. Currently, when a bundle is attached to the tangle and it is confirmed, it is possible to attach the same bundle again. If the bundle is still “valid”, it can be confirmed again. To further elaborate how this attack is performed, consider an address (sender) that owns some IOTA and want to transfer certain IOTAs to another address (receiver). When creating a transaction to do this, the sender must give “source and destination” addresses for the funds to be transferred. Thus, the sender creates a transaction that takes sender address as a source and receiver address as a destination for the IOTAs to be transferred and possibly another destination address for the remainder of the funds. If the sender decides to reuse the current address and does not specify an address for the remainder of the funds, then the remainder of the funds will be left at current address of the sender. The receiver could then “catch” that bundle containing the transaction of transferred IOTAs to receiver and replay it until sender address is drained. Every time the bundle, and thus the transaction, is replayed, the specified IOTA is sent from sender address to receiver address. In this case the owner of receiver address was able to steal IOTAs from sender address. Joseph Rebstock introduced this attack and explained a few variants [55]. He showed that when one address is reused, all addresses “downstream” of that address can be used by an attacker to drain the funds of the reused address. Draining funds from an address using downstream addresses is done through so-called “chain replays”. The replay vulnerability is also proved and exploited in [57].

**Figure 7:**
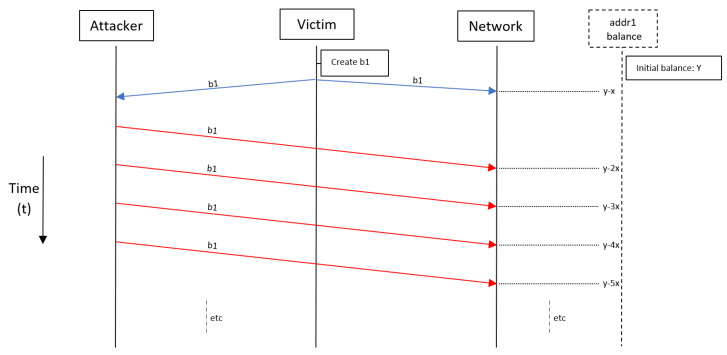
Replay attack (b1 is a bundle that transfers x IOTA from addr1 to addr2 and keeps the remaining balance back at addr1, thus addr1 is reused and vulnerable to replay attack).

Of course, for replaying, the attack is limited to existing bundles. However, if a reused address does not contain enough funds for a transaction to be replayed, the attacker may decide to top up the reused address. That will enable the attacker to replay the transaction and steal funds and also get back the funds he used to top up, which makes the cost of the attack equal to zero [55]. Figure 7 visualizes what happens in a replay attack. The victim creates a bundle that reuses a source address. The attacker notices this and starts to replay this bundle until the funds from the reused address are drained.

#### 3.2.1. Variants of Replay Attack

There are different ways to execute the replay attack. The simplest way is somehow making an IOTA user create a bundle that reuses their address. Then the attacker should catch and replay that bundle. However, there are also other ways to exploit the replay vulnerability.

##### Forcing Address Reuse

There are various ways IOTA users may be forced to reuse their addresses, possibly without even knowing it. For example, IOTA wallets (malicious) or other software (malicious) that uses the IOTA API, may force a user to reuse address. If a user just provides a destination address and a transfer amount, the software will create the bundle based on that information. The software may then create an address reusing bundle and send that into the network and in that way force address reuse. The problem with this approach is that it probably will be known if software reuses addresses when creating bundles. This is because software that uses cryptocurrency or facilitates cryptocurrency transactions in general needs to be open source to be accepted and adopted by users. This is because users want to know that they can trust the software they use, they want others and themselves to be able to review it. If the software is open source, code analysis will reveal that addresses are being reused, and as soon as that is known users will probably stop adopting the software.

In the unlikely scenario where the software code somehow was not analyzed well, users used the software for transactions and address reusing transaction bundles were created, it is still not likely that the attacker is able to force many address reuses. This is because visualizers like the https://thetangle.org (accessed 3 January 2021) allow the user to check bundles and see their inputs and outputs, then address reuse will also be visible. Furthermore, as soon as address reuse is discovered, users will likely abandon the address reusing software. If an attacker were to use software to force users to do address reuse, the attacker would first have to make users adopt the software. This will likely not succeed if it is known that the software reuses addresses. Thus, an attacker may first try to get a user base for the software, and then issue an update that introduces address reuse. In that way an attacker might be able to cause some address reuses but still it will be likely that the address reuse will be discovered soon, and upon that users will most likely abandon the software.

Another way to cause address reuse as an attacker is creating the transaction bundle that does address reuse yourself. Then send that (unsigned) bundle to the victim and somehow get the victim to sign it and send it into the network. Although this is theoretically possible (with the IOTA API), it is quite unlikely that a user will sign transactions that were made by someone else. Therefore, this way of causing address reuse also does not seem likely to succeed. Possibly if this is embedded in the way some payment system that uses IOTA works, it might be feasible, but it is likely to be discovered and thus not likely to succeed. Assuming that the attacker does not have direct access to the victim’s software, getting users to reuse their addresses is hard, and this makes the replay attack hard to execute in this way. Other variants, in which you do not have to force a user to reuse their address first might be more viable.

##### Brute Force

To create transaction bundles with the IOTA API, a user only needs a seed which has addresses with funds and the user needs to provide a destination address and a value. What an attacker might do is try to create transactions for a guessed seed. For example, suppose an address is owned by the attacker and certain transfer value of IOTA. The attacker may do the following:

- generate a random seed
- create an address reusing transaction bundle for that seed, transferring IOTA to attacker address
- if successful: replay the resulting transaction bundle until the addresses from that seed are drained. Else: go to step 1

It is questionable whether this will be worth it; the probability that a user finds a seed that contains funds is very low. This depends, among other things, on how many seeds are in use. Currently a seed is 81 Trytes, which means there are 27^81^ possible seeds, how many seeds are in use cannot be known. However, since there is such a large number of possible seeds, there seems to be a very small probability that an attacker actually finds a seed with nonzero addresses. However, it has been shown that multiple seeds can be used to access the same addresses, such that it may not be necessary to check all these seeds [66]. Further research needs to be done on whether brute force generating transactions for seeds is a viable way to create bundles that are vulnerable to replay attack. Currently it seems unprofitable because of estimated low probability of finding a used seed.

##### Past Transaction Bundles

According to [55], there have been transaction bundles already that reuse the input address. These bundles, if there is still IOTA left on the input address(es), are still vulnerable to the replay attack. Thus, an attacker may search through the tangle and try to find bundles that did address reuse, check the input address(es), and if there are still funds on them they can execute a replay attack. This is even stronger; the attacker can try to find addresses that have nonzero balance and has been used as an input for a transactions bundles before. Such addresses are vulnerable to the replay attack because the IOTAs that are still on these addresses can be deducted by replaying the bundles, if necessary after topping up the addresses. This means that any address that has been reused at least once (in the past) is vulnerable to the replay attack. An attacker may find these addresses and drain their funds. This variant of the attack still relies upon transactions that somehow reused an address. These may be hard to find and will at some point be drained as well. Then to keep the attack “usable”, the attacker should again try to force address reuse, which is hard as discussed above.

In response to the report about replay attacks IOTA stressed that address reuse is not supported and insecure, and thus should not be done. The IOTA Foundation is not planning on making replay attack impossible for now, because they say it is not a vulnerability, referring to the fact that addresses are not to be reused [67,68]. You can only become victim to this attack if you reuse your address, so if you use software that ignores IOTA’s recommendation against address reuse. However, the main reason that address reuse is discouraged is that an attacker may be able to force the private key for an address if it is reused. This is because so-called Winternitz One-Time signatures [69] are used for signing transactions, which become feasible to forge when an address is used more than once [70]. Furthermore, the replay attack vulnerability report claims that address reuse has been done already, and this attack has been executed [55].

### 3.3. Double Spending

A double spending attack, of which various variants exist, is an attack in which the attacker aims to spend the same funds multiple times. To illustrate how double spending works, consider an address (source) having some IOTAs and that spends all the funds in a transaction to another address (destination). After a while the transaction will be accepted and confirmed and as a result the balance of source address is 0. Now the source address issues second transaction sending some IOTAs to a different destination address. This should not be possible because the balance of source address is 0; however, if the attacker somehow achieves that this second transaction also gets confirmed by the network, the attacker was able to spend the funds twice. The challenge here is to get the second transaction accepted over the first transaction even though the first transaction was accepted earlier. In general, this exploit is executed by the attacker in the following steps:

1. spend a certain amount of IOTA in a transaction and publish that transaction
2. wait until the transaction is confirmed
3. publish a new transaction, conflicting with the previous through spending the same funds. The new transaction is the double-spending transaction
4. somehow make sure the new transaction gets accepted and confirmed such that the original legitimate transaction will be discarded, and everyone accepts the new transaction as legitimate transaction

The main challenge for the attacker lays in step 4, making sure the network accepts the double-spending transaction as the legitimate transaction, even though they already accepted the original transaction, which they should now discard. In the IOTA white-paper (v1.4.3) various ways to achieve this are described [14].

#### 3.3.1. Large Weight/Outpace Attack

**Figure 8:**
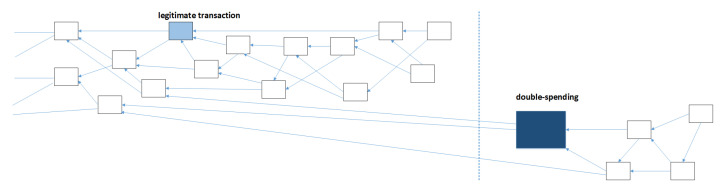
Large weight attack. (Inspired by [14]).

The way to get the fraudulent double spending transaction accepted is by making the other nodes select the tangle branch that contains this false transaction as the main tangle. Then the nodes will select tips on that branch to be approved by new transactions and such the double spending transaction will be accepted and confirmed; furthermore, the original (legitimate) transaction and its branch will be discarded. To have the nodes select the false branch, that branch should be more likely to be selected by the tip selection algorithm. In case the tip selection algorithm is based on cumulative weight, the false branch should have more weight than the honest branch to be more likely to be selected. The attacker therefore wants to give the false branch a lot of weight, and it could do this by doing a large weight attack. In a large weight attack, the attacker gives the double spending transaction on the dishonest branch a very large weight. If the weight is sufficiently large, nodes will choose the dishonest branch for future transactions; this is visualized in Figure 8.

The attacker may start preparing this large weight transaction in the time it takes for the legitimate transaction to be accepted. The probability that the attacker can generate a transaction with a larger weight than the weight of the honest branch is depending on the acceptation time, the attacker’s computing power and the weight growth rate of the honest branch. The probability that the attacker succeeds is non-negligible, and even if the attacker does not succeed in the acceptation period, they may keep trying after that. Then the attacker has a small but constant probability to succeed in “outweighing” the honest branch as time progresses such that the attack will at some point succeed [14]. Thus, the large-weight attack may always succeed; this can be shown as follows. The probabilities that the attack succeeds in the acceptation period and after were computed in [14]. These computations are explained to show that the large weight attack can always succeed. First some terms should be defined:

$W^{\left(n\right)}$: time to calculate weight of at least $3^{\left(n\right)}$

μ: attacker’s computing power

$w_{0}$: minimum weight a transaction needs to get to be accepted

$t_{0}$: time at which a transaction reaches weight bigger than or equal to $w_{0}$, where $t_{0}=0$ is the time at which the transaction was issued.

λ: the arrival rate of (honest) transactions.

*w*: average weight of an incoming transaction

⌈x⌉: smallest integer bigger than or equal to x

Now the probability that the attacker can generate a weight larger than or equal to the cumulative weight of the honest transaction in the acceptation time should be computed. Thus, the probability that “the time the attacker needs to generate the large weight transaction, with sufficiently large weight, is smaller than or equal to the time necessary for the honest transaction to be accepted by the network”. The time to generate the large weight transaction is expressed using $W^{\left(n\right)}$. Sufficiently large weight is weight bigger than or equal to the cumulative weight of the honest transaction after the acceptation time ($t_{0}$) has passed. The IOTA whitepaper [14] assumes $W^{\left(n\right)}$ is an exponentially distributed random variable. The parameter of its probability distribution is $\mu 3^{\left(-n\right)}$. We know that λ is the arrival rate of transactions and *w* is the average weight, such that λw is the average growth rate of the cumulative weight. Any transaction in the tangle has a cumulative weight growth bounded by λw; they reach this rate when they are (in)directly referenced by all tips. Thus the (maximal) cumulative weight of the honest branch after the acceptation period $t_{0}$ can be computed. This total weight ($w_{1}$) is equal to the maximal cumulative weight of the honest transaction after $t_{0}$ time, which is equal to $w_{1}=\lambda wt_{0}$. Now to succeed the attacker should, in time $t_{0}$, generate a transaction with weight $3^{n}\geq w_{1}$. This means that to get sufficient weight the condition $n\geq log_{3}\left(w_{1}\right)$ should be met. This is true for $n_{0}=\left\lceil log_{3}\left(w_{1}\right)\right\rceil$, because then $3^{n_{0}}\geq w_{1}$. Thus, the attacker needs to generate weight $3^{n_{0}}$ in time $t_{0}$. This means that $W^{\left(n_{0}\right)}$ should be smaller than $t_{0}$. Because it is given that $W^{\left(n\right)}$ is an exponential random variable with a parameter, the distribution function is known and the probability $P[W^{\left(n_{0}\right)}<t_{0}]$ can be computed. The cumulative distribution function for an exponential distribution is used to compute this probability. Then, in [14], the following formula results:

(1) $$P\left[W^{\left(n_{0}\right)}<t_{0}\right]=1-exp\left(-t_{0}\mu 3^{\left(-n_{0}\right)}\right)$$

The IOTA whitepaper [14] states that when $w_{1}$ is large, $3^{n_{0}}$ becomes about equal to $w_{1}$, which is used to simplify (4) as below. This assumption may be flawed. (This seems like an unrealistic assumption, comments on this are made later in this report)

(2) $$P\left[W^{\left(n_{0}\right)}<t_{0}\right]\approx 1-exp\left(-t_{0}\mu 3w_{1}^{-1}\right)$$

Then it is stated that $P\left[W^{\left(n_{0}\right)}<t_{0}\right]\approx \frac{t_{0}\mu }{w_{1}}$, with the comment that this is true in the case where $\frac{t_{0}\mu }{w_{1}}$ is small, which is a reasonable assumption [14]. Thus, the probability that the attack succeeds is largely dependent on the ratio between the computing power of the attacker and the network transaction rate, because $w_{1}=\lambda wt_{0}$. The given formulas apply to the initial phase, where the attacker tries to get a larger weight in the period it takes for the honest transaction to be accepted. After the honest transaction gets accepted the attacker may keep trying to outweigh it, by trying to get a weight of $3^{n}$ for $n>n_{0}$. The attacker may be successful when at the moment they get that weight, the honest branch has a smaller weight. At that moment the honest branch has weight $\lambda w\cdot W^{\left(n\right)}$, so the probability for the attacker to succeed is:

(3) $$P\left[\lambda wW^{\left(n\right)}<3^{n}\right]=1-exp(-\mu 3^{\left(-n_{0}\right)}\cdot \left(\frac{3^{\left(n_{0}\right)}}{\lambda w}\right)=1-exp\left(-\frac{\mu }{\lambda w}\right)$$

Which is then stated to be about equal to $\frac{\mu }{\lambda w}$. This is a constant probability that exists for every n, and because of that it can be assumed that the attack will most likely succeed at some point [14]. In addition, the attacker may start producing the large weight transaction well before issuing the legitimate transaction to the network. Then, when the acceptation time is over, and the attacker issues the double spending transaction, they had more time to generate a larger weight and thus they have a higher probability of succeeding in outweighing the honest branch. This leads to the conclusion that a large weight attack could always succeed. Therefore, countermeasures are necessary, which could be limiting the max weight of a transaction or setting the weight of a transaction to a constant. The IOTA whitepaper [14] also examines the situation were the maximum weight of a transaction is 1. Then the attacker still needs to generate more cumulative weight, using many nonsense transactions, than the rest of the network. It is found that it is necessary for the rate of honest transactions to be high compared to the computing power of the attacker, or else the probability that the attacker succeeds becomes non-negligible [14]. Currently the own weight of a transaction is the same for all transactions in the IOTA implementation, thus as long as the rate of honest transactions is large relative to the computing power of an attacker this attack will not succeed. In addition, the coordinator may prevent such an attack [14].

#### 3.3.2. Success Probability of Large Weight Attack

In the IOTA whitepaper (v1.4.3) [14] section about the large weight attack, a formula is given to compute the probability that the large weight attack succeeds. In the previous section it was noted that the assumption *“when $w_{1}$ is large, $3^{n_{0}}$ becomes about equal to $w_{1}$”*, is a possibly incorrect assumption. This will be further analyzed in this section, to discover the impact this may have on the success probability of the large weight attack. First, the given formula for computing the probability of success of the large weight attack is explained. Secondly, the assumption is analyzed, and the potential mistake in the assumption is discussed. Thirdly, the effect on the probability computations is scrutinized and the impact of the results is discussed.

The initial phase of the large weight attack is the phase that lasts until the original transaction gets confirmed. In this phase the attacker has the time to prepare a large weight transaction, and if this transaction has sufficient large weight the attack may succeed. By reasoning explained in the previous section, the probability of success for the attacker is expressed with the following formula [14]:

(4) $$P\left[W^{\left(n_{0}\right)}<t_{0}\right]=1-exp\left(-t_{0}\mu 3^{-n_{0}}\right)$$

Now for simplification the assumption is made that *"when $w_{1}$ is large, $3^{n_{0}}$ becomes about equal to $w_{1}$"*, and then the above formula is rewritten to:

(5) $$P[W^{\left(n_{0}\right)}<t_{0}]\approx 1-exp\left(-t_{0}\mu w_{1}^{-1}\right)$$

The terms in this assumption are defined as follows:

$w_{1}=\lambda wt_{0}$

$n_{0}=\left\lceil log_{3}\left(w_{1}\right)\right\rceil$

**Figure 9:**
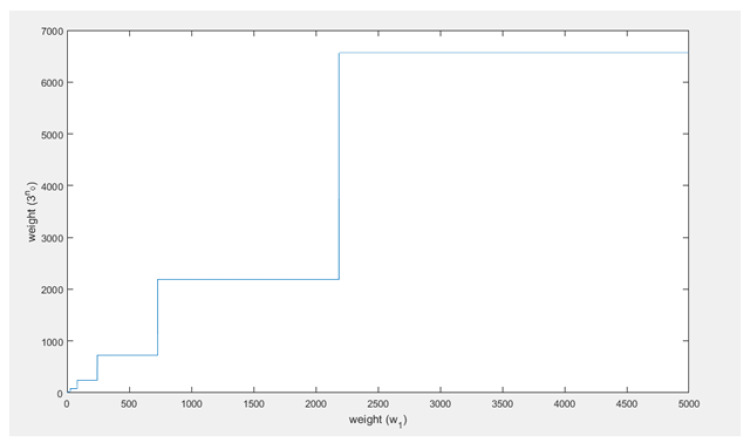
$3^{n_{0}}$ with respect to $w_{1}$.

**Figure 10:**
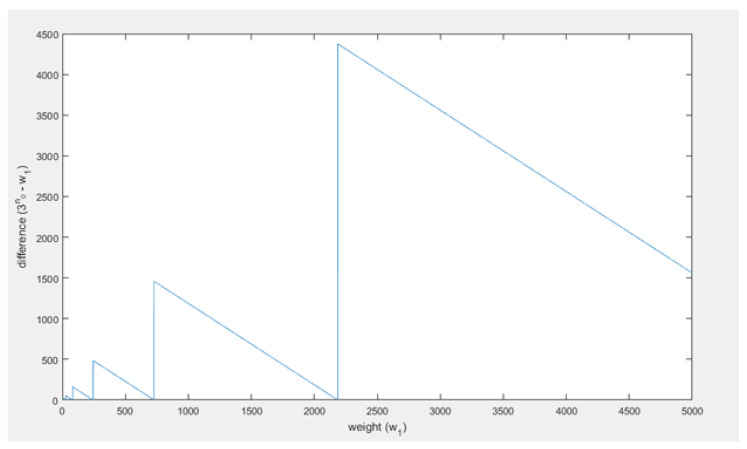
Difference between $3^{n_{0}}$ and $w_{1}$.

Thus, when the weight that the honest branch has gained after the confirmation time of the honest transaction $\left(w_{1}\right)$ becomes large, the weight that the attacker has to generate $\left(3^{n_{0}}\right)$, will become about equal to $w_{1}$. In fact, without the ceiling function which is used to compute $n_{0}$, $3^{n_{0}}$ is equal to $w_{1}$: $3^{log_{3}\left(w_{1}\right)}=w_{1}$. However, because of the ceiling function this equality is not true, in fact the difference between $3^{n_{0}}$ and $w_{1}$ will be variable over time but not converge or go to a stable difference, such that the assumption seems wrong. Below $w_{1}$, $3^{n_{0}}$ and the difference between them have been visualized. First, $w_{1}$ and $3^{n_{0}}$ are plotted in the same figure in Figure 9, then the difference is plotted with respect to $w_{1}$ in Figure 10.

From Figure 9 it can be concluded that, as $w_{1}$ increases, $3^{n_{0}}$ increases as well but in a different fashion. Furthermore, $3^{n_{0}}$ is always larger than $w_{1}$.

**Figure 11:**
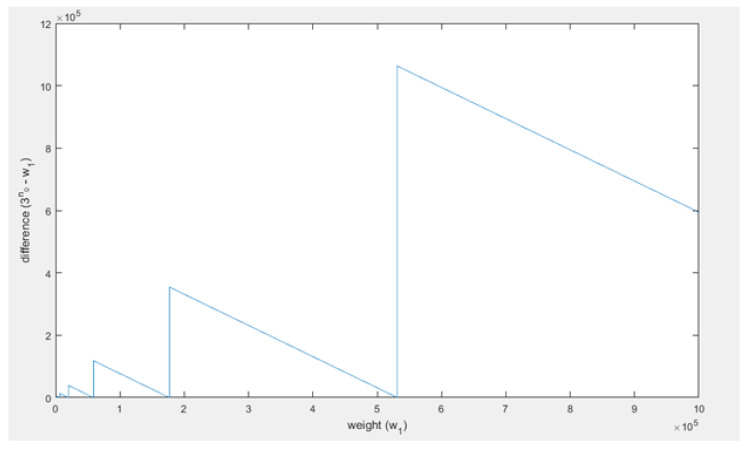
Difference between $3^{n_{0}}$ and $w_{1}$ as $w_{1}$ becomes very large.

It can be seen (Figure 10) that the difference does not converge or go to a stable difference. The difference between $w_{1}$ and $3^{n_{0}}$ behaves in a periodic fashion. It periodically becomes 0, but as *t* increases the period and amplitude increase as well. This is all due to the ceiling function used to compute $n_{0}$. This causes $3^{n_{0}}$ to always be a power of 3, whereas $w_{1}$ can be any (integer) value. Thus, as an example, when $w_{1}=28$, then log⁡28≈3.03 and thus $n_{0}$ will be 4. Therefore, then $3^{n_{0}}$ will be about 3 times $w_{1}$, and that will result something close to the maximal difference for that $w_{1}$ as well. The limit on the difference between $w_{1}$ and $3^{n_{0}}$ is equal to $2w_{1}$. Then as $w_{1}$ increases towards the next power of 3, the difference decreases to 0 and becomes zero when $w_{1}$ is equal to the next power of three. Right after that the difference will be close to $2w_{1}$ again. This oscillation repeats until infinity. Based on this analysis, $3^{n_{0}}\approx w_{1}$ as $w_{1}$ becomes large seems to be an irrational assumption. Even when $w_{1}$ becomes very large this pattern does not change as can be seen in Figure 11.

Although the assumption does not seem rational, this does not necessarily pose a problem. Next, the impact of this assumption on the probability computations will be elaborated.

The original formula, without the assumption, to compute the probability that the large weight attack succeeds is given in (4), and the probability formula including the assumption is given in (5). From here on these formulas will be referenced as P1 and P2, respectively. First the formulas will be rewritten to give more insight in the role that $3^{n_{0}}$ and $w_{1}$ play in the different formulas.

(6) $$P1=1-exp(-\frac{t_{0}\mu }{3^{-n_{0}}})$$

(7) $$P2=1-exp(-\frac{t_{0}\mu }{w_{1}^{-1}})$$

From the analysis earlier, it was found that periodically $3^{n_{0}}$ is much larger than $w_{1}$. In case that happens that would mean that $\frac{t_{0}\mu }{3^{n_{0}}}$ is much smaller than $\frac{t_{0}\mu }{w_{1}}$. It should also be noted that $t_{0}$ (time) and μ (measure for computing power) will have positive values, thus $\frac{t_{0}\mu }{3^{n_{0}}}$ and $\frac{t_{0}\mu }{w_{1}}$ will be positive values as well. Given $f=e^{-a}=\frac{1}{e^{a}}$, then if *a* is large, *f* will become small and vice versa, if *a* is small, *f* will become large. If we apply this principle to P1 and P2, where $\frac{t_{0}\mu }{3^{n_{0}}}$ is much smaller than $\frac{t_{0}\mu }{w_{1}}$, the result will be that P1<P2. Thus, in case the assumption is applied, the computed probability that a large weight attack succeeds is estimated larger than when the assumption is not applied. This also becomes visible when both P1 and P2 are visualized with respect to $w_{1}$. To generate these plots values for μ and $t_{0}$ were picked; however, any positive values can be used because these variables do not affect the trend.

**Figure 12:**
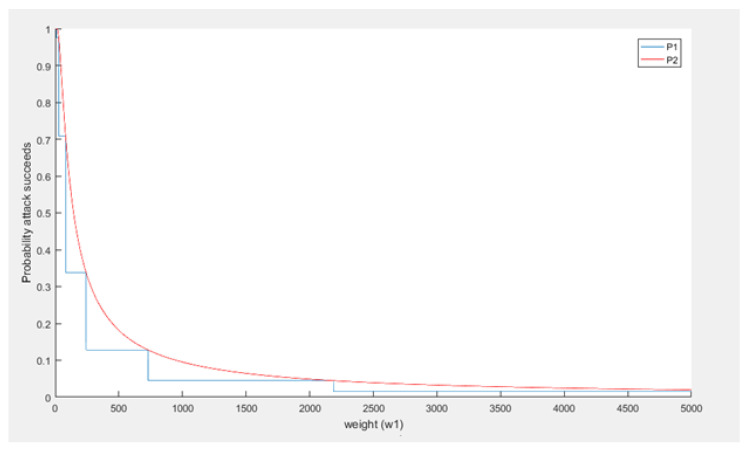
Probability of successful replay attack with respect to weight.

**Figure 13:**
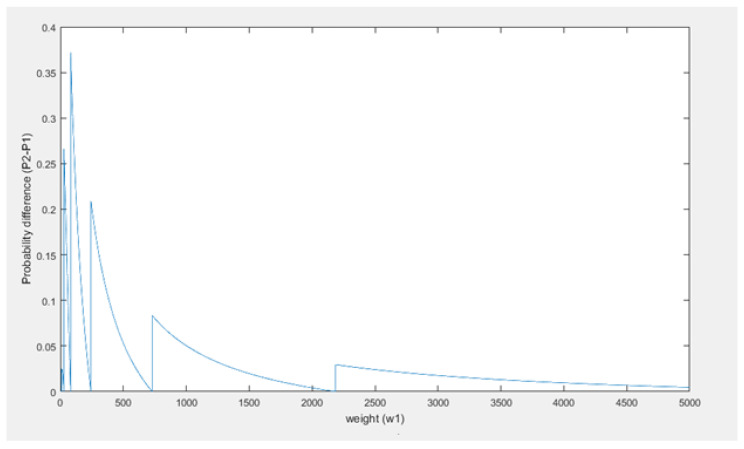
Difference between probabilities with and without assumption, with respect to weight.

In Figure 12, it can be seen that indeed P1<P2, thus the assumption does not positively influence the success probability computations. The difference between the probabilities has also been plotted in Figure 13. As shown, the difference becomes smaller as $w_{1}$ becomes bigger, in fact it goes to 0 as $w_{1}$ goes to infinity.

It can be concluded that a curious assumption is made in the IOTA whitepaper (v1.4.3) to simplify the computations for the probability that this attack succeeds. However, this assumption does not significantly influence the resulting probability computations. The final formula from the whitepaper which includes the assumption results in higher probabilities for success of the large weight attack than the formula that does not use the assumption. Based on the figures, using the assumption in the probability formula creates an upper limit on the actual probability, such that P2 is an upper limit of P1. Therefore, using P2 instead of P1, even if the assumption is curious and not true, does not underestimate the success probability and, therefore, is not necessarily wrong. In fact, it does give a nice result.

#### 3.3.3. Parasite Chain

**Figure 14:**
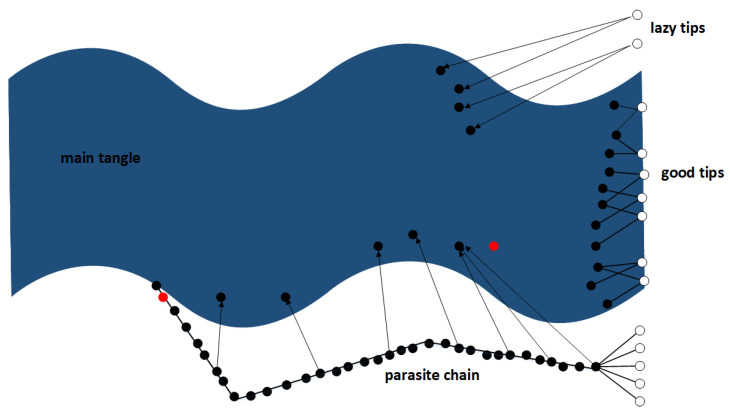
Parasite chain attack. (Adapted from [14]).

In the case the tip selection algorithm that is used is not based on weight but on height or score, the attacker may employ a different strategy to get the false branch selected by the nodes in the network. To issue a double spending transaction with a high score or large height in the tangle, the attacker may secretly create a so-called parasite chain, as shown in Figure 14. This is a chain of transactions that is not published to the network and that is built up by the attacker using a series of (nonsense) transactions. These transactions all reference each other in a chain like fashion, and some of them reference transactions on the main tangle. This parasite chain starts with the double spending transaction and references transactions on the main tangle only before the original transaction. Obviously, this parasite chain does not give a significant increase in the cumulative weight of the double spending transaction compared to the main tangle, because it is a chain of transactions. However, the attacker can artificially increase the number of tips at the end of its chain. In that way tip selection strategies that use random tip selection from the available set of tips also have a higher probability of choosing tips from the dishonest chain. In addition, tip selection algorithms based on height/score will have a higher probability for choosing tips from the dishonest chain, because the attacker can generate larger height and score by adding more transactions to the chain. The attacker can publish the parasite chain as soon as the legitimate transaction is confirmed [14].

Whether the parasite chain may be effective largely depends on the tip selection algorithm. The parasite chain can have higher values for height and score, but if cumulative weight is used the parasite chain does not have an advantage. This is also what is currently done in the MCMC tip selection algorithm. To select a tip, the algorithm places a “random walker” somewhere on an interval of the tangle. The walker then walks towards the tips in a pseudorandom fashion. The probability that a walker moves to one of the children of the current site depends on the cumulative weight difference between the current site and the child site. The smaller this difference, the larger the cumulative weight of a child and the bigger the probability that a walker moves to that child. The probability that the walker moves from x to y (thus y approves x), where the cumulative weight of x is H_x_ and the weight of y is H_y_, can be defined as follows. Assuming that the own weight of transaction sites is equal to 1, and α is a parameter that can be given, and z:z ⇒ x means all sites z that approve x, the probability can be computed [14]:

(8) $$P_{xy}=exp\left(-\propto \left(H_{x}-H_{y}\right)\right){\left(\sum_{z:z\to x}exp\left(-\alpha \left(H_{x}-H_{z}\right)\right)\right)}^{-1}$$

This algorithm prevents parasite chains from being successful; a parasite chain cannot contain as much cumulative weight as the main tangle unless the attacker has access to the majority of the (active) computing power in the IOTA network [14]. Thus, currently because the tip selection algorithm is used that is not based on height or score, the parasite chain is not a viable way to get a dishonest branch accepted by nodes in the IOTA network.

#### 3.3.4. Lazy Nodes

Next to parasite chain protection, the tip selection algorithm also offers protection against so-called lazy nodes in the network. A lazy node is a node that validates the same transactions over and over again. The tips generated by this lazy node will rarely be chosen by the MCMC random walk algorithm to be confirmed, because the algorithm prefers to “walk” to sites with a higher cumulative weight. Since the tips of the lazy node confirm sites that are much “earlier” in the tangle, they will have less cumulative weight than the other sites that confirm current tips and to which the “walker” could also “walk”, so there is a high probability they will not be confirmed [14].

### 3.4. Splitting Attack

Another attack that was presented in the IOTA whitepaper [14] is the Splitting Attack. In this attack an attacker splits the tangle by issuing two conflicting transactions. Then the attacker tries to maintain the balance between the two branches of the tangle [14]. Other nodes in the network may choose one of the two branches for confirmation when adding a transaction to the tangle. They cannot confirm transactions from both branches simultaneously because the branches are conflicting. If the amounts of transactions that are appended to each branch are roughly equal, the attacker may be able to maintain the balance between the branches, such that they will both grow and after some time both the conflicting transactions will be accepted and confirmed. In that way the attacker would be able to spend their funds twice. The attacker needs to maintain the balance only until the transactions have been confirmed.

In the IOTA whitepaper it is suggested that a “sharp-threshold” rule can help to prevent the splitting attack [14]. A sharp-threshold rule would make it very hard for the attacker to maintain the balance between branches in the tangle. When somehow the tangle is split in two branches, the honest nodes need to pick a branch when trying to confirm transactions. Which of the branches are picked by a node depends on the tip selection algorithm. In the MCMC algorithm this depends on the weights of the branches; the heavier branch will be picked with a higher probability. When a sharp threshold rule is implemented, the probability that the heavier branch is chosen is much larger than the probability that the lighter branch is chosen. Thus, even when there is a small total weight difference between the branches, still almost all honest nodes will pick the heavier branch. When all new transactions come in on one branch, the attacker will have to compensate a lot, which makes maintaining the balance, and thus the attack, much harder. A different defense mechanism is having a node publish a lot of transactions at once on one branch, such that there is a sudden big difference in total weight of the branches, this again means that the attacker must compensate a lot which is hard. In general, the splitting attack is considered hard because of the asynchronicity of the nodes: not all nodes necessarily have the same tangle. Because of that there may be many transactions the attacker does not know of and thus cannot (yet) compensate for [14].

**Figure 15:**
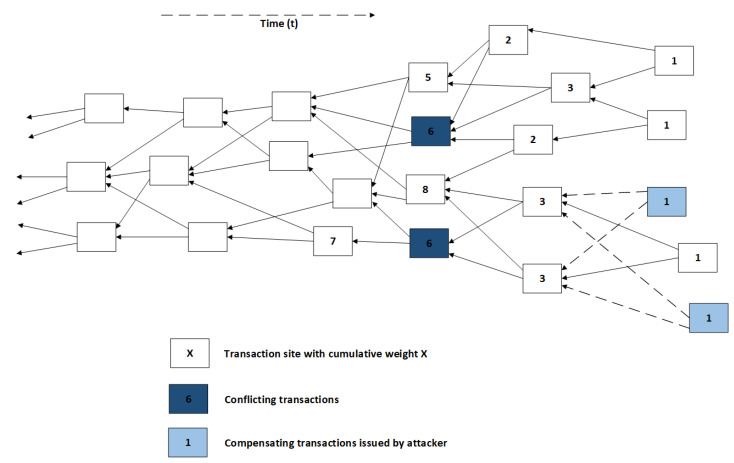
Splitting attack (tangle growth after splitting, when the branches become unbalanced due to incoming transactions the attacker balances them issuing own (nonsense) transactions).

In Figure 15 the splitting attack is visualized. The conflicting transactions split up the tangle in two branches, then as soon as the tangle becomes unbalanced the attacker issues transactions to rebalance the branches.

### 3.5. 34% Attack and Necessity of an “Assiduous Honest Majority”

A known attack in blockchain technologies is the 51% attack. This attack entails that the attacker somehow acquires the majority of the computing power in a network. Then the attacker is then able to impose his own rules to the rest of the network. The IOTA network is also vulnerable to an attack like that and it is repeatedly called the 34% attack [71,72,73]. In IOTA, this attack has slightly different requirements, namely a certain percentage of the computing power, a certain percentage of “omnipotence” and preferably knowledge of what the network topology looks like to optimally execute the attack [72]. The omnipotence of a node is its connectivity to the network, thus to how many other nodes in the network it is connected. The higher the percentages of total computing power and omnipotence, the more influence the attacker gets in the network. When the attacker is sufficiently “strong” they can do double spends and splitting, and with that threat they will be in control of the network. Earlier it was thought that the tip selection algorithm could offer some protection against this and other kinds of double spending attacks. However, it was proved that whatever tip selection algorithm you use, it is necessary to have a majority of active honest nodes in the network. That is, as long as there exists a so-called “maximal deterministic” version of the used tip selection algorithm, there is a way for the attacker to create an “optimal” sub tangle which will eventually outpace the honest tangle [74]. However, because currently the coordinator is being used, there is protection against attacks like this [71].

### 3.6. Centralization

The current implementation of the IOTA network (IOTA white-paper v1.4.3) has been called centralized [54,56], even though it is claimed to be decentralized [71]. The argument made for IOTA’s centralization is the existence of the coordinator. The coordinator, also called Coo, is a node in the IOTA network which plays a central role. Currently it is used to determine whether transactions are valid or not, to allow creating snapshots which are generated so that not every node has to store the full tangle history and to protect against certain types of attacks. Even though the IOTA Foundation claims that the nodes could do without the coordinator and that IOTA is decentralized, it does seem that the Coo has some power over the network. IOTA foundation response to centralization claim is that the Coo is a temporary solution. It is a protection against 34% attacks and other attacks, which is needed in the “infancy stage of the tangle” [71]. The role of the Coo is said to be analogous to that of training wheels; it is planned to be shut down in the future. Eric Wall [56] gives several arguments that show that IOTA is currently at least somewhat centralized. Firstly, he notes the issue of validating transactions. A transaction is confirmed only if it is referenced (in)directly by a milestone transaction. The milestone transactions are transactions generated by the coordinator, thus in IOTA, consensus about the validity of a transaction is reached through the confirmation of a central unit. Then secondly, this brings another risk, if somehow someone is able to get the coordinator’s private key, they can produce unlimited milestone transactions and then the purpose of the coordinator is gone. Thus, there is a “single point of failure”, which makes IOTA seem more centralized. Thirdly, the Coo is said to offer protection against various types of attacks, thus for security of the IOTA network there appears to be another central point of failure.

## 4. Impacts and Prevention of IOTA Vulnerabilities

In this section the impacts and prevention of the six vulnerabilities we have identified are discussed.

### 4.1. Curl-P Hashing Collisions

This vulnerability would allow an attacker to apply the signature that a user applied to a certain transaction to a different transaction. This vulnerability was caused by the hash function IOTA used. The vulnerability was denied by the IOTA foundation, and to prevent this attack IOTA updated their hash function to a hash function without known collisions. Therefore, whether attacks based on this issue were viable or not, currently they are not viable anymore because the hash function has been replaced. Thus, this vulnerability currently does not seem to pose a threat. Research remains to be done on whether the other purposes, such as PoW, of this hash function cause vulnerabilities as well.

### 4.2. Replay Attack

In the replay attack the attacker sends a transaction that already has been confirmed into the network again, and then it will get confirmed again. In that way the transaction will be executed multiple times. This vulnerability relies on address reuse. Assuming the attacker does not have access to the victim’s software, it will be hard for the attacker to force victims to reuse their addresses. Thus, even though the replay attack is technically feasible, the replay vulnerability does not seem to pose a large threat. However, the variants of the attack do not all require to force victims to reuse their address. Therefore, to be more secure, IOTA could find ways to refuse address reusing transactions. The impact of a successful replay attack depends on the address that was reused. All the funds that are left on the address after it has been reused are vulnerable to the replay attack. In addition, addresses that have been reused a long time ago, but still contain funds, are vulnerable to the replay attack. In that sense the impact of this vulnerability is large, because all reused addresses are affected.

The replay attack can be prevented in various ways. In [55] it is suggested to “keep track of the unique hash of each signed transaction bundle”, and then only allow one instance of a bundle hash in a subtangle. To do that bundle hashes would have to be made unique, no collisions should occur, and they should be sufficiently long such that a virtually unlimited number of bundles can be created. In addition, each node should then check each incoming transaction bundle and compare their hash with a database of known bundle hashes of the current subtangle. Then the node should only accept the transaction bundle if it has a hash that does not exist yet. Another prevention mechanism might be to make (signed) transaction hashes unique and allow only an individual instance of each transaction hash in a subtangle. In that case transaction hashes would have to be unique and unlimited in availability. Both of the prevention mechanisms described above will likely cause quite some overhead. When the IOTA network grows the amount of incoming transactions will increase and thus the database storing the used hashes will grow and the time to maintain this database and check incoming transactions will likely increase.

### 4.3. Double Spending

Two ways to do a double spending attack in IOTA have been discussed. In the current IOTA implementation only the large weight attack has potential. No specific prevention mechanism is required against this attack since the parasite chain attack is not viable because IOTA does not select the main subtangle based on height or score but on cumulative weight, and the parasite chain is just aimed at getting higher score/height. Furthermore, IOTA is currently protected against a large weight attack as it is described because the own weight of a transaction is limited, such that the attacker cannot generate one transaction with a very large weight. In addition, it is shown using mathematics that the probability that the large weight attack succeeds is small. Moreover, the Coordinator protects against such attacks.

### 4.4. Splitting Attack

The splitting attack is an attack in which the tangle is split into two conflicting subtangles. The attacker can then spend their funds on both subtangles. To get the transactions on both subtangles confirmed, the attacker should maintain the balance between the subtangles. Then the incoming transactions will divide themselves between the subtangles, and such transactions on both subtangles will gain confirmation. Through that, even the conflicting transactions will be confirmed. Many preventive measures against this attack have been suggested. Primarily, it is hard for the attacker to maintain the balance between two branches, and this can be made even harder by randomly adding “bursts” of transactions to one of the subtangles and maintaining a sharp threshold. The sharp threshold will cause all incoming transactions to attach to the largest subtangle. Thus, as soon as the balance between the subtangles is disturbed a little bit, it will be very hard for the attacker to restore it. Furthermore, if the network is large enough, and the transaction rate is relatively high compared to the amount of computing power of the attacker, it will be impossible for the attacker to maintain the balance. Although this attack is technically hard to execute, if it were executed it would significantly impact the security of the IOTA network.

### 4.5. 34% Attack and Necessity of an “Assiduous Honest Majority”

The 34% attack is the IOTA variant of the blockchain 51% attack. This attack has slightly different properties and requirements but not much is known about the attack. To execute the attack the attacker needs some percentage of the computing power and sufficient connectivity to the rest of the network. If successful, the attacker will be able to impose their rules on the IOTA network. Currently the coordinator is part of the IOTA network, and it prevents such attacks. Even though currently not a viable attack, more research is necessary to understand this attack and to evaluate its potential when the coordinator is removed.

### 4.6. Centralization

IOTA has been called centralized because of the role of the coordinator node in the IOTA network. This issue is not a direct vulnerability to IOTA but a common criticism of IOTA. The network uses a coordinator, which is a node in the network that has power over the network because it issues milestone transactions. Milestone transactions are necessary for transaction confirmation, thus indirectly the coordinator decides whether a transaction will be confirmed, and thus there is no real distributed consensus. This is judged as central power and it creates a single point of failure. Centralization is commonly seen as a security risk for distributed ledger technologies like cryptocurrencies. IOTA Foundation claims that IOTA is decentralized. It is stated that the IOTA network could function without the coordinator as well, but the coordinator is also said to offer protection against some attacks. It is questionable whether IOTA can function without the coordinator at all, because that has not been proven. Even though this is not an immediate vulnerability, it is an indirect IOTA security issue. This security issue can be prevented by removing the coordinator. However, future research should evaluate whether IOTA can function without a coordinator, such that there is distributed consensus as well.

## 5. Threat Modeling

In this section the CVSS v3.0 [19] framework is discussed and implemented to characterize the six IOTA vulnerabilities mentioned earlier. The aim is to characterize the threats posed by these vulnerabilities. Threats are potential risks to the security measures implemented. Threat modeling is a process that helps to accurately evaluate and perceive the attack surface, assign risk to the threats and drive the vulnerability mitigation process [75]. The significance of threat modeling is to improve the overall security.

**Figure 16:**
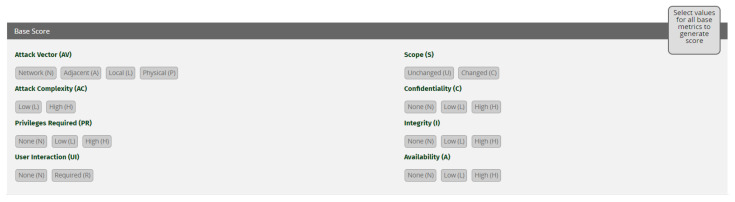
Base metric group with metric names and values.

**Figure 17:**
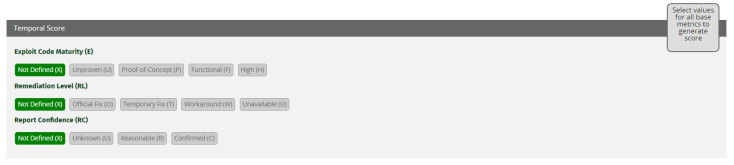
Temporal metric group with metric names and values.

CVSS v3.0 [19] consists of three different scoring models (metric groups): base, temporal and environmental [76]. Base metrics constitute attributes of vulnerabilities that are incessant with time and the network. The temporal metrics represent attributes of vulnerabilities that can change over time and may need to be adjusted. The temporal metrics shows the state of a vulnerability and the availability and confidence of exploitation mechanisms. The Environmental metric group represents the vulnerabilities that are specific to certain network or architecture [19,77]. Base and temporal metric group include metric names and metric values. Severity score depends on the metric values selected in the metric names. Samples of base and temporal metric groups are shown in Figure 16 and Figure 17. The higher the score of the metric group, the more severe the vulnerability is. Among these three models, we have implemented base scoring and temporal scoring models. Environmental metric group is excluded since we are characterizing these vulnerabilities more generically rather than for a specific network or environment. For detailed mathematics behind the severity calculation, please refer to CVSS v3.0 [19] specification document [78].

According to [19,77,78], in base metric, attack vector (AV) demonstrates the environment (network, local, physical) where vulnerability can be exploited. Base score increases if the vulnerability can be exploited remotely. Attack complexity metric represents the circumstances that the attacker can use for his advantage to exploit a vulnerability although the attacker has no control over these circumstances, for example presence of certain configuration settings. Lesser the complexity to exploit a vulnerability higher the base value. Privileges required metric expresses the level of privileges an attacker requires in order to exploit a vulnerability. If no privileges are required to exploit a vulnerability, then the base score is the highest. User interaction metric describes the victim role in the exploitation of the vulnerability. The base score is greater if no victim interaction is necessary. Scope metric describes the impact of the vulnerability beyond the exploitable device or software. The base score is greater when impact affects systems beyond the vulnerable component. Confidentiality metric indicates the effect on the data confidentiality after a vulnerability is exploited by an attacker. The base score increases with the degree of data disclosed. Integrity metric demonstrates the impact on the integrity of data if a vulnerability is exploited. Base score increases with the degree of information that can be modified for malicious purposes. Availability metric describes the impact on the availability of a device or network caused by vulnerability exploitation. The base score increases with the degree of resource availability affected.

As stated in [19,77,78], in temporal metric, exploit code maturity computes the plausibility of a certain vulnerability being exploited and the current state of the available exploit techniques. The temporal score increases when the exploit is easily available and can be executed by unskilled users. Remediation level shows the type of remediation available; it illustrates the various stages of patch availability for a vulnerability. Report confidence illustrates the actual availability of the vulnerability and the technical details related to the vulnerability. Temporal score increases if the vulnerability is known and validated to exist with certainty. Temporal metric has default value of “Not defined” and is used when no sufficient information to select a particular metric value or do not want to use the metric. “Not defined” produces the same value as worst case metric value. For both the metric groups, qualitative severity rating scale [79] is performed using CVSS v3.0 calculator [76]. Textual representation is mapped with numeric base, such as: none (0), low (0.1–3.9), medium (4.0–6.9), high (7.0–8.9) and critical (9.0–10.0). It is important to note that as vulnerabilities and patches are evolving quickly, the metrics’ values also evolve over time.

### 5.1. CVSS v3.0 Base Score

The following is the base score calculated for all the six vulnerabilities. The scoring is based on our analysis and the literature and information available at the time of performing threat modeling. With different interpretations and outlook the scores might be disparate.

#### 5.1.1. Base Score for Curl-P Hashing

**Figure A1:**
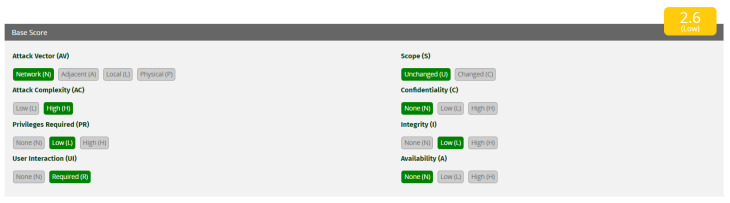
Base score for Curl-P hashing.

For Curl-P hashing, the attack vector is network because it is exploitable remotely. A successful attack requires conditions beyond the attacker’s control so the attack complexity is high. As the attacker is an authorized user, but has no control over the the other users’ components, we chose privileges (public, private keys) required as low. User interaction is required. The exploited and impacted component is the same, so the scope is unchanged. Confidentiality impact is none, because no confidentiality is lost. In this vulnerability either the attacker is trying to steal or waste funds so there is lost of integrity only in the sense of funds. The attacker cannot modify all the information/data on the victim account so the integrity impact is low. This vulnerability has no impact on the availability. The overall base score for Curl-P hashing is low (2.6) as shown in the Appendix A Figure A1.

#### 5.1.2. Base Score for Replay Attacks

**Figure A2:**
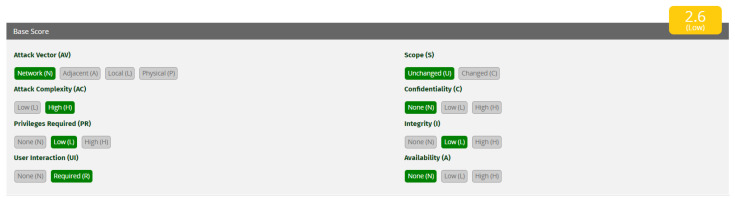
Base score for replay attack.

This attack is exploited remotely so the attack vector is network. In general, executing replay attack is highly complex, so attack complexity is high. As all the users are authenticated (public and private keys) in IOTA, the attack does not require privileges to control over the victim, thus the privileges required is low. User interaction is required and the scope is unchanged. Replay attack has no impact on confidentiality and availability; however, as funds can be wasted or stolen by replaying there is low impact on the integrity. For replay attack the overall base score is low (2.6) as shown in the Appendix A Figure A2.

#### 5.1.3. Base Score for Double Spending

**Figure A3:**
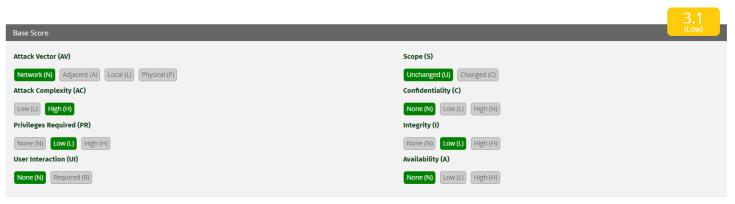
Base score for double spend attack.

Furthermore, double spending attack is also performed remotely, thus the attack vector metric is network. Successful attack complexity is high, as it requires significant technical efforts from the attacker. This vulnerability can be exploited with low level privileges. No user interaction is required, and successful exploitation cannot further be nurtured to exploit other components in the IOTA network so the scope is unchanged. This exploit has no impact on confidentiality and availability; however, the impact on integrity is low. The overall base score for double spending attack is low (3.1) as shown in the Appendix A Figure A3.

#### 5.1.4. Base Score for Splitting Attack

**Figure A4:**
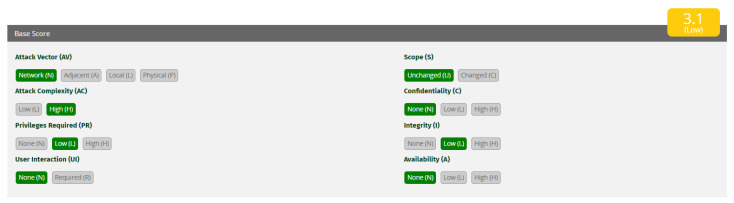
Base score for splitting attack.

The attack is executed remotely, hence attack vector is network. Furthermore, the attack is complex to perform and maintain. As the attacker is authorized (owns public, private keys) in the tangle, the privilege required is low. The scope remains unchanged in splitting attack. Confidentiality and availability is not impacted. The affect on integrity is low if funds are lost. The overall base score for splitting attack is low (3.1) as shown in the Appendix A Figure A4.

#### 5.1.5. Base Score for 34% Attack and Necessity of an “Assiduous Honest Majority”

**Figure A5:**
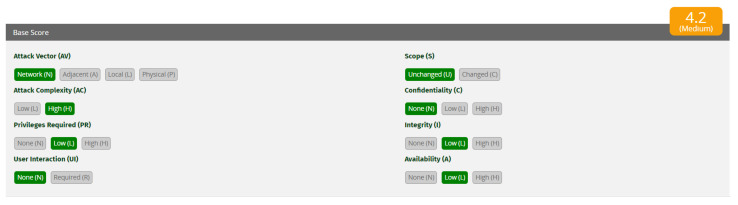
Base score for 34% attack and necessity of an “assiduous honest majority”.

Similar to the previous attack, the attack vector is network, the attack complexity is high, the attacker is authorized (owns public, private keys) in the tangle, no user interaction is required, integrity impact is low, the scope is unchanged and confidentiality is not affected. Except in this attack, the attacker is able to impose his own rules on the rest of the network so there can possibly be adverse impact on the performance of nodes or network resources, hence impact on the integrity and availability is low. The overall base score for this vulnerability is medium (4.2) as shown in the Appendix A Figure A5.

#### 5.1.6. Base Score for Centralization

**Figure A6:**
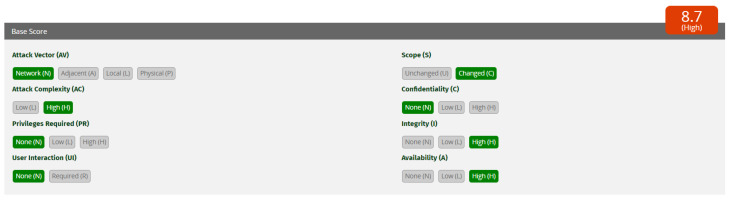
Base score for centralization issue.

The centralization issue of IOTA can be exploited remotely, exploiting the attack is highly complex, no privileges might be required (for example, performing denial of service against coordinator), no user interaction is required, as coordinator is acting as central authority scope might change if successfully exploited, and confidentiality might not be impacted; however, the integrity and availability might be highly impacted. The overall base score for centralization issue is high (8.7) as shown in the Appendix A Figure A6.

The Base scores for all the six vulnerabilities are recapitulated in the Table 1.

### 5.2. CVSS v3.0 Temporal Score

The following are the temporal scores calculated for the six IOTA vulnerabilities.

#### 5.2.1. Temporal Score for Curl-P Hashing

**Figure A7:**
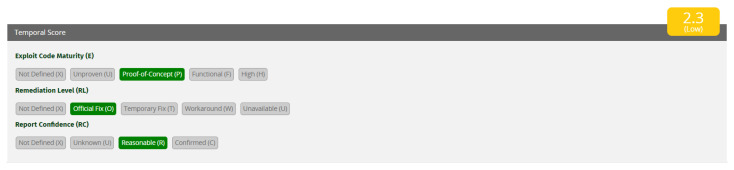
Temporal score for Curl-P hashing.

The exploit code maturity value is proof-of-concept because this vulnerability is exploited in a very naive use case and requires substantial efforts from skilled attacker. The IOTA foundation responded to this vulnerability by updating the hashing function thus the remediation level is official fix. The report confidence for this vulnerability is reasonable because a detailed exploitation report is available with a proof-of-concept. However, even though the IOTA foundation did issue a fix for the hashing collisions, they maintain the position that the vulnerability never posed a security threat and that they were aware of its existence. Overall temporal score is low (2.3) as shown in the Appendix B Figure A7.

#### 5.2.2. Temporal Score for Replay Attack

**Figure A8:**
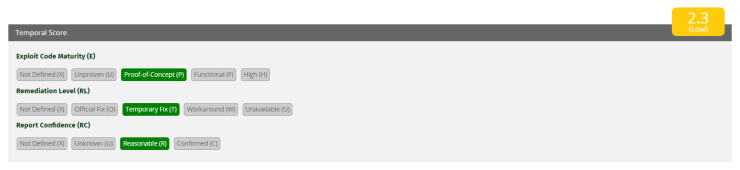
Temporal score for Replay attack.

In order to successfully exploit replay attack, proof-of-concept exploit code is available but requires modification in IOTA API from a user perspective, and substantial efforts are required from a skilled attacker. Therefore, exploit code maturity is proof-of-concept. Following IOTA’s official usage recommendations avoids the replay attack vulnerability, because it discourages address reuse. Still, the remediation level is temporary fix because the user can still reuse (intentionally or unintentionally) addresses. The complete fix would be if the address reuse is invalidated. Vulnerability report confidence is reasonable. Overall score is low (2.3) as shown in the Appendix B Figure A8.

#### 5.2.3. Temporal Score for Double Spending

**Figure A9:**
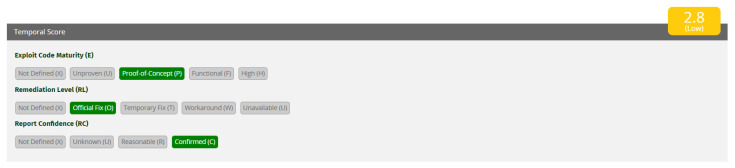
Temporal score for Double spending.

Exploit code maturity for double spending is proof-of-concept, remediation level is official fix and report confidence is confirmed, because this vulnerability is discussed and prevention is provided in IOTA whitepaper (V 1.4.3) [14]. Overall temporal score is low (2.8) as shown in the Appendix B Figure A9.

#### 5.2.4. Temporal Score for Splitting Attack

**Figure A10:**
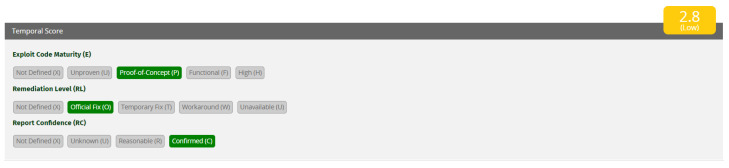
Temporal score for Splitting attack.

This vulnerability is also discussed and remediation is provided in the IOTA whitepaper (V1.4.3) [14]. Therefore, exploit code maturity metric value for splitting attack is proof-of-concept, remediation level is official fix and report confidence is confirmed. Overall temporal score for this vulnerability is low (2.8) as shown in the Appendix B Figure A10.

#### 5.2.5. Temporal Score for 34% Attack and Necessity of an “Assiduous Honest Majority”

**Figure A11:**
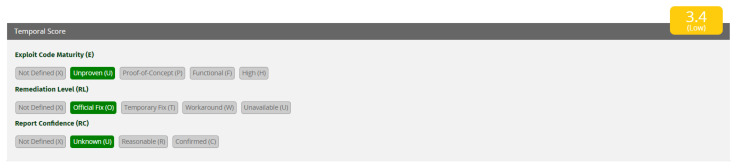
Temporal score for 34% attack and necessity of an “assiduous honest majority”.

This vulnerability is theoretical, hence exploit code maturity is unproven. Remediation level is official fix because IOTA foundation claims coordinator can prevent this attack. This vulnerability is not practically exploited and there is little confidence in the validity of the reports, thence report confidence is unknown. Overall temporal score is low (3.4) as shown in the Appendix B Figure A11.

#### 5.2.6. Temporal Score for Centralization

**Figure A12:**
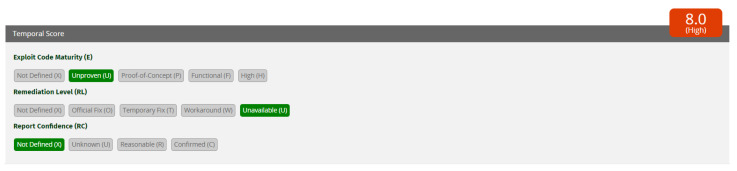
Temporal score for Centralization.

Centralization appears to pose a significant threat to IOTA; however, it is not practically exploited and the impact is unknown. Therefore, exploit code maturity is unproven and report confidence is not defined. And presently remediation is unavailable. Final temporal score for centralization is high (8.0) as shown in the Appendix B Figure A12.

Temporal scores for all the six vulnerabilities are shown in the Table 2.

## 6. Discussion on Threat Model

Vulnerabilities studied in this work are quite drastic and if successfully exploited can stimulate hindrance in the mass acceptance and establishment of IOTA in many application domains. However, from Table 1 and Table 2, it is vivid that most of these vulnerabilities pose low severity threats. From our analysis and understanding there could be multiple reasons for the low severity. For example, IOTA technology itself is rather new and complex and is rarely adopted; therefore, more efforts are required to explore and exploit it. Furthermore, the lack of publicly available exploits drags the severity scores to lower proportion. If, in the future, IOTA becomes mainstream technology, attackers can and will develop new exploitation methodologies which will require revising the severity.

## 7. Conclusions

This report presents an overview of IOTA security issues mainly found online and in the literature. Vulnerabilities described in this report are some of the most commonly known vulnerabilities, and the IOTA foundation is aware of their existence and somehow acknowledges them. IOTA is an emerging technology and has demonstrated its potential use in various application domains. However, its security is not yet fully explored and scrutinized. Therefore, its vulnerabilities and patches are not yet so mature. CVSS is used to characterize the identified vulnerabilities in order to communicate the characteristics and severity of the vulnerabilities. We have evaluated two important metric groups: base and temporal of CVSS. Each of these groups consists of a set of metrics. These sets of metrics are analyzed, explained and interpreted for each of the six identified vulnerabilities. It is concluded that IOTA could be made more secure by mainly fixing issues like the replay attack and the centralization. However, based on this research we have inferred that, with respect to the discussed vulnerabilities, IOTA is secure if used according to the recommendations from IOTA foundation. Moreover, the analyzed vulnerabilities currently do not pose any significant threat to overall IOTA security. Still, more research into the individual vulnerabilities, and into other potential vulnerabilities, is necessary to discover their potential impacts.

## 8. Future Research

Currently proof of work is essential in IOTA; this might be limiting for IoT devices since IoT devices have low computational and storage capabilities. Future research is required to investigate IOTA viability for usage by IoT devices and whether IoT devices can be active participants of the IOTA network. Outsourcing proof of work could be a solution, but then it should be researched whether IOTA would become centralized because of that. Furthermore, as noted in this report, IOTA does seem centralized and it is unclear whether the IOTA network would be able to securely function without the coordinator. Therefore, research into the viability of the IOTA network without the protective coordinator and with fully distributed consensus should be done.

## Figures and Tables

**Table 1:** Base scores for the six vulnerabilities identified.

Vulnerability	CVSS Score	Rating
Curl-P hashing	2.6	low
Replay attack	2.6	low
Double spending attack	3.1	low
Splitting attack	3.1	low
34% attack and necessity of an “assiduous honest majority”	4.2	medium
Centralization	8.7	high

**Table 2:** Temporal scores for the six vulnerabilities identified.

Vulnerability	CVSS Score	Rating
Curl-P hashing	2.3	low
Replay Attacks	2.3	low
Double Spending	2.8	low
Splitting attack	2.8	low
34% attack and necessity of an “assiduous honest majority”	3.4	low
Centralization	8.0	high

## Data Availability

Not applicable.
